# Characterization of Serum Proteins Associated with *IL28B* Genotype among Patients with Chronic Hepatitis C

**DOI:** 10.1371/journal.pone.0021854

**Published:** 2011-07-05

**Authors:** Derek D. Cyr, Joseph E. Lucas, J. Will Thompson, Keyur Patel, Paul J. Clark, Alexander Thompson, Hans L. Tillmann, John G. McHutchison, M. Arthur Moseley, Jeanette J. McCarthy

**Affiliations:** 1 Institute for Genome Sciences and Policy, Duke University Medical Center, Durham, North Carolina, United States of America; 2 Duke Clinical Research Institute and the Division of Gastroenterology, Duke University Medical Center, Durham, North Carolina, United States of America; 3 Division of Community and Family Medicine, Duke University Medical Center, Durham, North Carolina, United States of America; University of South Florida College of Medicine, United States of America

## Abstract

**Introduction:**

Polymorphisms near the *IL28B* gene (e.g. rs12979860) encoding interferon λ3 have recently been associated with both spontaneous clearance and treatment response to pegIFN/RBV in chronic hepatitis C (CHC) patients. The molecular consequences of this genetic variation are unknown. To gain further insight into *IL28B* function we assessed the association of rs12979860 with expression of protein quantitative traits (pQTL analysis) generated using open-platform proteomics in serum from patients.

**Methods:**

41 patients with genotype 1 chronic hepatitis C infection from the Duke Liver Clinic were genotyped for rs12979860. Proteomic profiles were generated by LC-MS/MS analysis following immunodepletion of serum with MARS14 columns and trypsin-digestion. Next, a latent factor model was used to classify peptides into metaproteins based on co-expression and using only those peptides with protein identifications. Metaproteins were then analyzed for association with *IL28B* genotype using one-way analysis of variance.

**Results:**

There were a total of 4,186 peptides in the data set with positive identifications. These were matched with 253 proteins of which 110 had two or more associated, identified peptides. The *IL28B* treatment response genotype (rs12979860_CC) was significantly associated with lower serum levels of corticosteroid binding globulin (CBG; p = 9.2×10^−6^), a major transport protein for glucocorticoids and progestins. Moreover, the CBG metaprotein was associated with treatment response (p = 0.0148), but this association was attenuated when both *IL28B* genotype and CBG were included in the model, suggesting that the CBG association may be independent of treatment response.

**Conclusions:**

In this cohort of chronic hepatitis C patients, *IL28B* polymorphism was associated with serum levels of corticosteroid binding globulin, a major transporter of cortisol, however, CBG does not appear to mediate the association of *IL28B* with treatment response. Further investigation of this pathway is warranted to determine if it plays a role in other comorbidities of HCV-infection.

## Introduction

Hepatitis C virus (HCV) infecton is a global health concern with an estimated prevalence of up to 170 million people infected worldwide [1]. Complications from liver disease as a consequence of chronic hepatitis C (CHC) infection is the leading cause for liver transplantation in the United States [2]. Response to current standard of care interferon-based treatment regimens is effective in less than one-half of infected individuals. Recently, polymorphisms in the vicinity of the *IL28B* gene, encoding interferon λ3, have been associated with both spontaneous clearance and virologic response to pegylated interferon-alpha and Ribavirin therapy (pegIFN/RBV) among patients with CHC [3]–[7]. The underlying functional genetic variant(s) responsible for this observed association remains to be identified, yet several SNPs, including rs12979860, appear to be robust markers of its effect.

CHC patients who respond to pegIFN/RBV tend to have lower pre-treatment expression levels of interferon stimulated genes (ISGs) in the liver [8]. Honda and colleagues carried out expression quantitative trait locus (eQTL) analysis, examining the *IL28B* SNP association with liver gene expression levels of ISGs and found that hepatic ISGs were significantly lower in HCV patients with the *IL28B* ‘responder’ genotype compared to patients without this genotype [9]. These results were independently replicated in another study [10], supporting the idea that a lower endogenous interferon response increases the likelihood of treatment response. However, little else is known about the mechanism through which *IL28B* influences viral clearance.

We recently assessed a cohort of CHC patients and reported a proteomic signature of HCV treatment response measured in pre-treatment serum samples that was highly predictive of response to peg-interferon/Ribavirin [11], with equivalent performance to *IL28B* genotype. Interestingly, the proteomic signature and *IL28B* genotype demonstrated only partial correlation, suggesting unique pathways to predict response. Serum proteomic data from that investigation were utilized in the current study to explore the correlation of genetic variation with downstream protein expression. This protein quantitative trait locus (pQTL) analysis builds on the success of eQTL analysis, where correlation of genetic variation with downstream mRNA expression has provided insights into the functional consequence of genetic variation. Prior studies employing similar methods have demonstrated the effectiveness of such an approach for identifying genetic variants controlling protein expression in yeast [12]. In humans, large-scale pQTL analysis of proteins measured by standard laboratory assays have also been performed [13]. Both studies confirm the utility of identifying both *cis*-acting (located close to the gene encoding the protein) and *trans*-acting (located elsewhere in the genome) loci. In the current study we use an unbiased, label-free proteomic platform to characterize serum proteins associated with an *IL28B* polymorphism that has been strongly associated with HCV clearance.

## Results

The study included 41 genotype 1 CHC patients that were predominantly Caucasian (78%), male (61%), treatment responders (63%) with moderate to advanced fibrosis (85%) and a mean age of 47.4 years. The favorable response *IL28B* CC genotype was observed in close to one-half of our cohort. Table 1 summarizes this patient cohort.

**Table 1:** Characteristics of the 41 Chronic HCV patients in the present study.

Characteristic	N (percent)
Race	
Caucasian	32 (78%)
African American	9 (22%)
Sex	
Male	25 (61%)
Female	16 (39%)
Treatment response	
SVR	26 (63%)
NR	15 (37%)
Rs12979860 genotype	
CC	20 (49%)
TC	18 (44%)
TT	3 (7%)
	Mean ± SD
Age (years)	47.4±6.1

### Metaproteins associated with IL28B genotype

**Figure 1:**
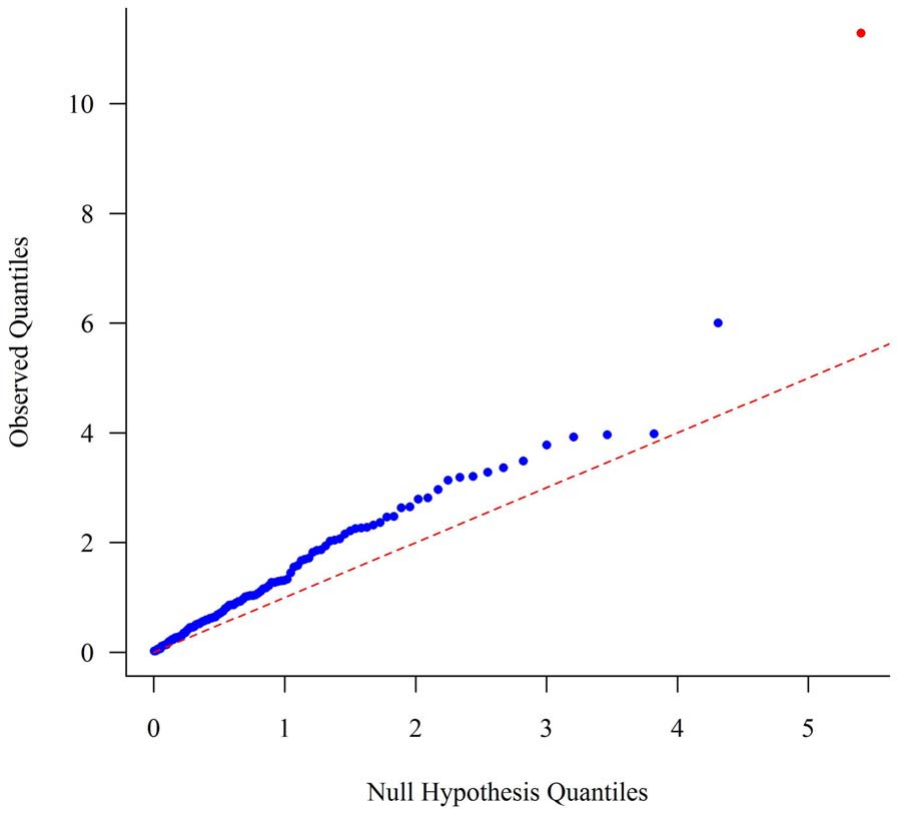
Quantile-Quantile plot showing the distribution of observed P values for *IL28B* rs12979860 genotype association with 110 metaproteins among chronic HCV patients to the expected distribution of P values under the null hypothesis. The red point denotes the CBG metaprotein.

**Figure 2:**
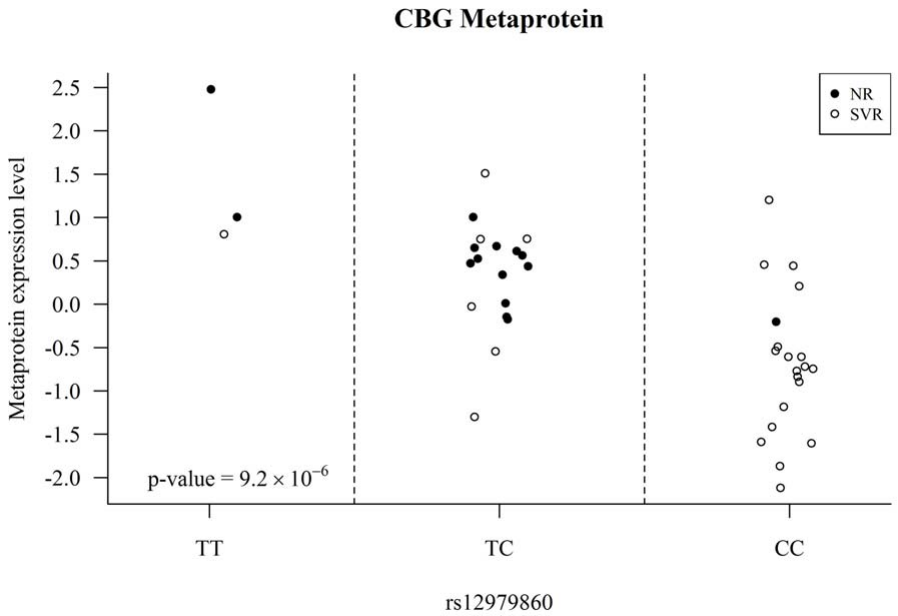
Expression level of CBG metaprotein by *IL28B* rs12979860 genotype in 41 Chronic HCV patients. Filled circles represent non-responders (NR) to interferon treatment and open circles are from subjects who achieved sustained virologic response (SVR) to treatment.

There were a total of 4,186 peptides in the data set with identifications by MS/MS at a 1% false discovery rate. Latent factor modeling grouped the individual peptides into 110 metaproteins. P-values generated from the association analysis of each of the 110 metaproteins and rs12979860 genotype are shown in the quantile-quantile (QQ) plot in Figure 1. A single metaprotein showed significant association with *IL28B* genotype after Bonferroni correction for multiple hypothesis testing: the liver protein corticosteroid binding globulin (CBG; p = 9.2×10^−6^). The rs12979860 ‘C’ (responder) allele was associated with lower levels of CBG metaprotein and explained 46% of the variance in CBG metaprotein (Figure 2). These results remained significant when testing a recessive model (CC versus TC/TT; p = 7.45×10^−5^).

If the previously reported association between *IL28B* and SVR is mediated through CBG, we would expect to see an association between CBG and SVR in this cohort as well. Indeed, we found a statistically significant association between the CBG metaprotein expression and SVR (p = 0.0148). We also found a statistically significant association (p = 0.0021) between the *IL28B* genotype and SVR, as reported previously in patients from the Duke Hepatology Clinic cohort [14]. However, when including both *IL28B* genotype and CBG in a model to predict SVR, the association with CBG was completely attenuated (p = 0.46) while the *IL28B* association remained significant (p = 0.0145), suggesting that CBG is probably not mediating the association between *IL28B* and SVR. These results are summarized in Table 2.

**Table 2:** Statistical associations.

Dependent Variable	Independent Variable†	Additional Covariates	Odds Ratio (95% CI) for Independent Variable	P-value for Independent Variable
SVR	CBG	sex, race	2.62 (0.93, 8.64)	0.0148
SVR	*IL28B*	sex, race	0.03 (0.001,0.19)	0.0021
SVR	*IL28B*	sex, race, CBG	0.05 (0.002, 0.39)	0.0145
			Beta estimate	
CBG	*IL28B*	sex, race	1.16	7.45×10^−5^

† For *IL28B* as the independent variable of interest, we are testing a recessive model, i.e., CC vs. TT/TC.

**Figure 3:**
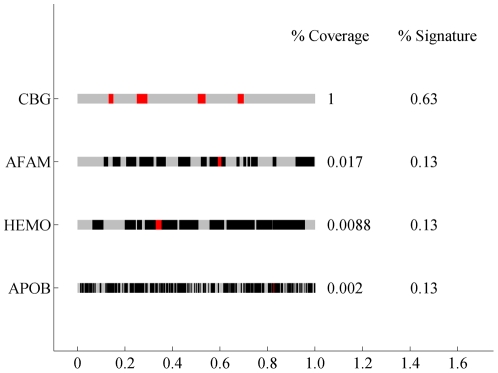
CBG metaprotein peptide composition. Each gray bar is a representation of the labeled protein. Red sections represent polypeptides from isotope groups that are in the factor and black sections represent polypeptides from the isotope groups that are identified in the data set but are not in the factor. Gray represents sections of the protein that are not identified in the data set. % Coverage is the percent of identified peptides from that protein that are in the factor and % Signature is the percent of the factor that comes from the associated protein.

The CBG metaprotein included all (5 of 5) of the CBG peptides generated, as well as individual peptides from three other proteins (Figure 3). As expected, the correlation between all five of the CBG peptides is high (0.84<r<0.95) and correlations between CBG peptides and the AFAM, HEMO and APOB peptides are also high (0.57<r<0.94). Each of these component peptides was tested for association with *IL28B* genotype to determine whether all or only some of the peptides contributed to the association observed with the metaprotein (Table 3). All eight component peptides were found to be statistically significant, even with correction for multiple hypotheses (p≤0.006).

**Table 3:** Association of component peptides from CBG metaprotein with *IL28B* genotype.

Peptide	Monoisotopic M/Z	Peak Centroid Time	Charge State	Peak Time Score	P-value
CBG	740.3704	68.17205,68.173,68.17549,68.17624,68.19092	2	0.8491636,0.8916217,0.9133328,0.95755863,0.9581282	1.46×10^−5^
CBG	717.356	58.446613,58.451298,58.462116,58.475937,58.496063,58.50195,58.507416	3	0.91022366,0.91063344,0.91442996,0.918305,0.93158853,0.93290806,0.9367805	1.50×10^−4^
AFAM	553.2891	32.656315,32.71019	1	0.9143975,0.9606747	1.74×10^−4^
CBG	538.30505	59.005672,59.028744	2	0.9508445,0.9678301	2.42×10^−4^
APOB	461.27652	32.52292,32.57379	2	0.8160564,0.96448535	3.40×10^−5^
CBG	432.76743	32.560402,32.605553	1	0.7805042,0.9042755	5.17×10^−5^
CBG	864.5479	32.484024,32.5972	3	0.8152123,0.9661816	6.36×10^−4^
HEMO	1238.0748	66.4398,66.45684,66.46535,66.47275,66.47417	2	0.80079937,0.80459476,0.8478435,0.88555866,0.8909334	7.37×10^−5^

## Discussion

We recently reported the successful application of an unbiased LC-MS/MS platform for proteomic expression signatures associated with HCV treatment response [11]. Using this proteomic data, we were able to demonstrate a relationship between protein expression and genetic variation at the *IL28B* gene locus, a strong determinant of treatment-induced HCV clearance. Corticosteroid binding globulin peptides were strongly associated with *IL28B* genotype, accounting for over a third of the variance of the CBG metaprotein and thus, *IL28B* may be an important *trans*-pQTL for serum CBG levels among HCV-infected individuals. We were unable to show that CBG mediates the relationship between *IL28B* and treatment response, indicating possible pleiotropic effects of this locus.

CBG, also known as Transcortin, is a major high-affinity plasma transport protein for glucocorticoids and progestins and is expressed primarily in the liver. Serum cortisol and CBG levels are inversely related [15], [16], thus, we would expect rs12979860-CC to associate with not only lower CBG but higher cortisol levels as well. Cortisol modulates the inflammatory response, and the primary function of CBG in proinflammatory states such as CHC infection is to deliver cortisol to sites of inflammation leading to down regulation of neutrophil activity and the cell mediated immune system. Treatment of CHC patients with interferon-beta has been linked to increases in both inflammatory cytokines and cortisol, but these effects appear to be independent [17]. Similarly, administration of interferon-alpha has been shown to alter plasma cortisol levels and immune cytokines, but these appear to be independent effects [18]. Based on our research, we postulate that the link between interferon and cortisol levels may hold for endogenous interferon lambda as well. Moreover, this relationship is unrelated to treatment response and thus, supports pleiotropic effects of interferons on the steroid hormone system and treatment response.

Levels of serum CBG may be altered in chronic disease states, but are unlikely to confound our results. CBG levels may be reduced in patients with advanced stage liver disease [19]; however, no patients in this cohort had evidence of impaired hepatic function or decompensated disease. Moreover, both the *IL28B* rs12979860-CC responder genotype and advanced liver disease areas associated with lower CBG levels, but *IL28B* rs12979860-CC is not associated with a lower rate, but rather a more rapid rate of hepatic fibrosis in CHC patients [20]. In addition, *in vitro* studies have demonstrated an inhibitory effect of insulin and insulin-like growth factor-1 on CBG mRNA expression in a human hepatoblastoma derived Hep G2 cell-line [21]. The inverse correlation between insulin and CBG has also been observed in small clinical studies [22] and upregulation of CBG has been proposed as a marker of type 1 diabetes mellitus [23]. The prevalence of insulin resistance is increased among patients with CHC infection and is associated with reduced virologic response to IFN-based therapy [24]. We did not specifically assess insulin resistance in our cohort. However, we found the *IL28B* rs12979860-C responder allele (and not the rs12979860-T ‘poor response’ allele that would be expected to associate with insulin resistance) to be associated with low serum levels of the CBG metaprotein.

Despite strong statistical support for the observed association between *IL28*B and CBG in our study, the results were based on a relatively small number of patients and should thus be considered preliminary. Post-pubertal CBG levels do not vary significantly with age or gender, and there is minimal diurnal variation, thus reducing the potential influence of normal physiological variation on our observations [25]. Moreover, the generalizability of these findings, including whether this is a mechanism specific to infection with HCV, or an association with a chronic inflammatory state is unknown. Replication in larger cohorts will be necessary to validate and extend these findings. Studies that incorporate direct measures of cortisol and CBG may provide further validation of the relationship between *IL28B* genotype and corticosteroids.

The current study utilized proteomic data generated by LC-MS/MS and analyzed using novel methods to group peptides into ‘metaprotein’ clusters. Thus, the intact proteins were not measured directly, but rather, specific groups of peptides representing the protein were measured. The metaprotein algorithm preferentially, but not exclusively, groups identified peptides according to their assigned “parent” protein. In addition, the algorithm permits exclusion of a peptide from the model if its expression trend does not track with the other peptides, and inclusion of peptides from different proteins or unidentified peptides which have expression in common with the metaprotein. In this way, future work correlating metaproteins with genes identified in pQTL analysis may assist in identification of currently unidentified proteins, or at least functional correlation of these unknown peptides with biological mechanisms.

In summary, the current study used an open-proteomic platform to characterize protein expression associated with a polymorphism strongly associated with HCV clearance and have identified a strong *trans*-acting pQTL, providing insight into the biological consequences of this variation. We have found the CBG metaprotein and its constitutent peptides to be strongly associated with *IL28B* genotype, but not mediating the effect of *IL28B* genotype on treatment response. Our research showcases the power of pQTL analysis for the functional annotation of genetic variants.

## Materials and Methods

### Patient population and ethics statement

Patient samples were selected from the Duke Hepatology Clinical Research (DHCR) database and were included in a prior validation study of proteomic signatures of virologic response to current IFN-based standard-of-care therapy [11]. The cohort included 41 patients with chronic genotype 1 HCV infection who had serum samples available prior to treatment, known treatment response, and DNA available for genetic analysis. All patients provided written informed consent for genetic testing and all study procedures were approved by the Duke University Institutional Review Board.

### *IL28B* polymorphism

The genomic region associated with HCV response in [4] contains several highly correlated SNPs around the *IL28B* gene. We selected the most strongly associated SNP, rs12979860, located upstream of this gene for genotyping in our cohort using the 5′ nuclease assay with allele specific TaqMan probes [26].

### Sample preparation and LC-MS/MS analysis

Sample preparation and LC-MS/MS analyses on these samples have been described previously [11]. Briefly, serum samples were immunodepleted using MARS14 columns and then subjected to in-solution digestion with trypsin. LC-MS/MS analysis was carried out on a nanoAcquity liquid chromatograph coupled to a QToF Premier mass spectrometer (Waters Corporation, Milford, MA). The Rosetta Elucidator® v3.3 software package (Rosetta Biosoftware, Rosetta Inpharmatics LLC, Seattle, WA) was used to import and align all LC-MS raw data files and perform feature quantitation. Mascot v2.2 (Matrix Sciences, Inc, Boston, MA) and Proteinlynx Global Server v2.4 (Waters Corporation, Milford, MA) database search engines were used to make peptide identifications.

### Metaprotein construction

Several challenges exist in the analysis of proteomic data generated by LC-MS/MS. Incorrect peptide identifications, sharing of peptides among homologs, and post-translationally modified or processed peptides that may show a biologically relevant and different expression pattern than the proteotypic peptides, can all lead to errors in the quantitative analysis of proteins. In order to correct for these multiple sources of noise, we utilized our metaprotein factor model which groups peptides based on co-expression. We restricted our analysis to only those peptides with protein identifications. Because co-expression across the biological cohort is a central tenant of this modeling approach, peptides are not exclusively tied to their parent protein and therefore individual peptides arising from multiple proteins may potentially comprise a single metaprotein. Metaprotein nomenclature was based on predominance of peptides associated with a known protein. Complete details of how metaprotein expression levels are generated as well as mathematical details regarding the metaprotein factor model are described in Document S1. Protein identification frequency in samples from the 41 CHC patients is listed in Table S1.

### Association analysis

Each metaprotein was analyzed for association with *IL28B* genotype using one-way analysis of variance (ANOVA). *IL28B* genotypes (TT, CT, CC) were analyzed assuming an additive model, controlling for sex and race. Subsequent analysis was carried out assuming a recessive model for the response allele (C), comparing CC to CT/TT genotypes, consistent with the effect of this locus on treatment response. All statistical analyses have been performed using MATLAB; a commented script (Materials S1) and workspace (Materials S2) have been included as supporting information.

## Acknowledgments

We gratefully acknowledge Martha Stapels and Scott Geromanos for assistance in LC-MS method development and data collection, Cindy Chepanoske and Andrey Bondarenko for assistance in raw data processing, Diane Uzarski, Crystal Cates and Melissa Spain for assistance with database and biorepository serum sample processing.

## Supporting Information

Document S1 Details of metaprotein expression level generation and metaprotein factor model construction.
(PDF)

Table S1 Protein identification frequency in samples from 41 CHC patients. This table lists each of the proteins identified in the data set along with the proteins' full names and the number of different isotope groups from that protein that were identified.
(DOC)

Materials S1 Matlab script.
(M) Click here for additional data file.

Materials S2 Matlab workspace.
(MAT)
