# Supplementary material for: Characterization of Serum Proteins Associated with IL28B Genotype among Patients with Chronic Hepatitis C
Source: PLoS One. 2011 Jul 5;6(7):e21854. doi: 10.1371/journal.pone.0021854 (PMC3130042; doi:10.1371/journal.pone.0021854)
Supplement: Document S1 — Details of metaprotein expression level generation and metaprotein factor model construction. (PDF) [file pone.0021854.s001.pdf]

Supporting Information

Following is an excerpt from:

Lucas et al., "Metaprotein Expression Modeling for Label-Free Quantitative Proteomics", *J. Proteome Res*. In review. Accepted for oral presentation, 2011 RECOMB Satellite Conf. on Computational Proteomics. March 11-13, 2011, San Diego, CA.

Sample Preparation

Serum samples were statistically randomized (3X) and the sample processing was performed blind to treatment group. Samples were thawed on ice and vortexed briefly. 50 uL of each serum samples was diluted 1:4 with Agilent immunodepletion Buffer A (5185-5990) and centrifuged through a 0.22 μm filter (5185-5990) at 10000 rpm. A 10 uL aliquot was removed for total protein assay (mini Bradford, Bio-Rad, Inc.), and the samples were pipette into glass LC vials and stored in the autosampler at 4° C until analysis. The Agilent Multi Affinity Removal Column HU-14, 4.6 × 100 mm (5188-6558), was installed on an Agilent 1100 HPLC system, and conditioned with five 20μL plasma depletions before the first sample injection (normal plasma from Bioreclamation, Inc.). HPLC-based immunodepletion and fraction collection was then performed as instructed by the manufacturer, except that only 20 ul serum equivalent (100 ul total) was injected on the column in order to ensure that the column had sufficient capacity to remove the target proteins across the sample cohort. The 1 mL unbound fraction, collected from 7 to 17 minutes during depletion, was desalted and buffer exchanged into 50 mM ammonium bicarbonate (EMD 1.01131.0500) at least 100x using a 10 kDa MWCO filter (Amicon 4, Millipore, Inc.). The final sample was concentrated to approx 50uL, after which protein concentration was measured with a mini-Bradford assay. A 5 ug aliquot of each sample was run on a Novex SDS-PAGE gel (Invitrogen, Inc.) as quality control measure. Approximately 20 ug of each sample was aliquoted for digestion, and sample concentrations were normalized to approximately 0.8 ug/uL with 50 mM ammonium bicarbonate (EMD). Following normalization, Rapigest SF (Waters, 186001861) was added to 0.1% w/v. Samples were then reduced, alkylated, and digested following a standard in-solution digestion protocol (http://www.genome.duke.edu/cores/proteomics/sample-preparation/). The samples were

reduced in 10 mM dithiothreitol (VWR, VW1506-02), alkylated in 20 mM iodoacetamide (Calbiochem 407710), and digested with 0.4 ug sequencing grade modified trypsin (Promega V5111). After digestion overnight, samples were acidified to 1% trifluoroacetic acid (Pierce, PI28904) and heated for 2 hrs at 60° C to remove Rapigest. Samples were then centrifuged at 15,000 rcf for 10 mins and the supernatant was pipetted into total recovery LC vials (Waters Corporation).

LC-MS Operation

Each sample was analyzed by injecting approximately 1 ug of total digested protein onto a 75um × 250 mm BEH C18 column (Waters) and separated using a gradient of 5 to 40% acetonitrile with 0.1% formic acid, with a flow rate of 0.3uL/min, in 120 minutes on a nanoAcquity liquid chromatograph (Waters). Electrospray ionization was used to introduce the sample in real-time to a Q-Tof Premier mass spectrometer (Waters), collecting data in MSE mode with 0.9 second alternating scans between low CE (6V) and a high CE ramp (15V to 40V). Data collection in this fashion supplies sufficient sampling across the chromatographic elution of a peptide for accurate quantitation, while also allowing acquisition of data used for the qualitative identifications. Technical reproducibility was assessed by running a subset of the samples in triplicate (n=6) and also by analyzing a pooled sample at predefined intervals. In addition, a number of data-dependent LC-MS/MS analyses were performed using the same LC gradient and injection volumes; these runs provided column conditioning prior to quantitative analysis, and in some cases complementary peptide identifications.

Preparation of data for analysis

To accomplish data alignment and feature quantitation across all biological samples and thus form the matrix discussed in the statistical methods section below, we utilized Rosetta Elucidator™v3.3 software package (Rosetta Biosoftware) to import and align all MSE and data-dependent acquisition (DDA) raw data files.[1-5] Database searches were performed against a forward/reverse Swissprot database (v 56.5) with human taxonomy, using ProteinLynx Global Server v2.4 (IdentityE algorithm,Waters Corporation) for MSE searches or Mascot v2.2 for DDA data. Database searches are either performed externally and results imported (PLGS 2.4) or queued directly from within Elucidator (Mascot) to allow identification of many of the

quantified features in the proteomic dataset. All database searches were performed with high mass accuracy on precursor and product ions (typically 20 ppm precursor and 0.04Da product ion tolerance), with fixed carbamidomethylation(Cys), variable oxidation(Met) and variable deamidation(Asn and Gln). Annotation of the peptides is accomplished at an estimated 1% FDR using the Elucidator implementation of PeptideProphet algorithm.[6] Visual scripting within Elucidator is utilized to extract feature intensities for those features which have quantitative values above the 1000 counts (approximately 10th percentile) in 50% of the samples. The final file for statistical analysis is made up of a matrix of intensities, with the rows corresponding to isotope groups and the columns to technical observations (LC-MS analysis). An isotope group is defined as all of the peaks associated with a single peptide at a specific charge state and retention time. This level of quantitation combines peaks from the same peptide that differ according to the number of carbon 13's incorporated, but does not combine the same peptide measured at different charge states. The intensity of an isotope group for a given sample is the total volume under the feature peaks associated with that isotope group. This is monotonically related to the concentration of that isotope group in the original sample, and it is these intensities that we work with.

Metaprotein Statistical Model

In order to estimate metaprotein abundance, we build our model from pre-processed data (described in previous section) with intensity estimates aggregated at the isotope group level. We introduce the term metaprotein here to differentiate this approach from those in which peptide identifications lead to a fixed assignment of a particular peptide to a protein. In our modeling approach, we allow the possibility that an isotope group will be incorrectly identified, or be correctly identified, but have a pattern of expression that is distinct from the bulk of peptides from the corresponding protein. In practice, this new grouping approach often leads to metaproteins which may be dominated by isotope groups from a particular protein, but which contain isotope groups from other proteins as well.

Let X be a $P \times N$-dimensional matrix consisting of measurements on P isotope groups across N samples.

$$X = \mu 1_N' + A\Lambda' + \varepsilon \tag{1}$$

The P-dimensional vector μ has elements μ_i representing the mean expression of isotope group i and 1_N is a column vector of ones. The $N \times K$-dimensional matrix Λ represents latent factors which will be learned from the data and A is a $P \times K$-dimensional matrix of factor loadings with elements $a_{i,k}$. The random variable ε is a $P \times N$ matrix of idiosyncratic noise.

Our goal is to estimate relative protein concentration from this model using the latent factors in Λ. Recall that we have identifications for some subset of the isotope groups. With this in mind, suppose we identify each column of A and the corresponding column of Λ with one identified protein. If we set $a_{i,k}$ = 1 when isotope group i is from a peptide identified as coming from protein k and $a_{i,k}$ = 0 otherwise, then our model is describing the expression pattern of each isotope group as a noisy approximation of the expression pattern of the protein, where the protein is known.

Retaining, for the time being, the idea of fixing $a_{i,k}$ in this way, we wish to handle the possibility of changing sensitivity and changing protein concentration from sample to sample. To account for this, we introduce an additional set of latent factors into equation 1.

$$X = \mu 1_N' + BH' + A\Lambda' + \varepsilon \tag{2}$$

We now introduce latent factors H and loadings $B = (b_{i,j})$ where $j = 1 \cdots J$ which we use to account for systematic structure in the data that is sample specific. Because these features will span almost all peptides, we utilize a generic Gaussian prior for the elements of B.

$$b_{i,j} \sim N(m_0, v_0)$$

This distribution represents our belief that these effects span all isotope groups, but with varying effect sizes. This prior also minimizes identifiability issues between B, which is not sparse, and A which is very sparse with somewhat informative priors.

We want to modify our prior on A to allow for possible post-translational modifications and for misidentifications. With this in mind, we want to relax our strict assignment of zeros and

ones in the loadings matrix A. Instead, our prior distribution for $a_{i,k}$ will reflect our level of certainty that we know which factor should represent the expression of this peptide. When we have an identification for peptide i and have mapped that peptide to protein k, our prior distribution will reflect an increased certainty that $a_{i,k} \neq 0$.

We introduce a p-dimensional vector of latent variables (z_i) which identifies the non-zero column of A for each isotope group. When we have an identification that suggests that isotope group i comes from protein k, our prior distribution for z_i is

$$z_i \sim Multinomial(1, q_i)$$
$$q_i \sim Dir(a_0, \cdots, a_0, a_k, a_0, \cdots, a_0)$$

where a_k is substantially larger than a_0 to reflect our prior belief that $z_i = k$. We default to $a_k = 500 \cdot a_0$, but have tried values from 100 through 1000 and these lead to only minor shifts in metaprotein membership. As the weight of this prior decreases (a_0 decreases while the ratio of a_0 to a_k stays the same) we are decreasing the importance of identification information and placing progressively more importance on correlation structure. We have found, for the Hepatitis application below (omitted from this document; available upon request), that the association of metaproteins with outcome doesn't substantially change until we increase the weight of the identification data to very high levels. We find that using $a_0 = 1$ leads to interpretable metaproteins without loss of association with the outcomes. For peptides which do not have identifications, we utilize an unbiased prior $z_i \sim Dir(a_0)$. Because different peptides showing similar expression patterns may, nonetheless, show a different magnitude of expression of that pattern due to the relative sensitivity of the mass spectrometer for the peptide, we model each of the non-zero elements of A independently, such that $a_{i,k} \sim N(m_a, v_a)$ when $z_i = k$ and $a_{i,k} = 0$ otherwise.

We note that, in the limit as $a_k \rightarrow \infty$ we obtain an ANOVA model in which there is no uncertainty about which metaprotein each isotope group belongs to. This is precisely the fixed effects, feature-specific variance model by Clough et al.[7] which implies that[7] is a limiting case of the model we present here. That limiting model implies that identifications are assumed to be accurate and assumes that post-translational modifications are of minor importance. Clough et al. correctly point out that, by collecting features one obtains higher power for detecting

associations versus simple tests of association with individual isotope groups. That model, as well as the one we present here, may be expanded with additional design vectors identifying experimental groups or particular interventions if so desired. These may be included as rows in H which are simply not updated in the MCMC.

To complete the model specification, we assume a conjugate, row specific inverse gamma prior for the variance of ε. This allows differing variance estimates for each isotope group. Because we are working with a relatively large number of samples (and thereby have no issues with identifying variance), we use a prior with mean 1 and variance 100. We also assume that the individual columns of Λ arise from a uniform distribution on the N-dimensional sphere of radius $\sqrt{N}$. The model is fit via Markov chain Monte Carlo (MCMC) and the result of this fit is a set of draws from the posterior distribution of all of the model parameters. All prior distributions are conjugate, and therefore we may use Gibbs sampling to update the model parameters at each step of the MCMC. The data sets we are modeling have been aggregated at the isotope group level, and as such they have between 20 and 40 thousand measurements per sample. While our sampling scheme is able to fit this data in just a few hours on a desktop, we expect that some sort of parallel processing will be desirable for data that is aggregated at the feature level. We have tested our model on multiple simulated data sets of various sizes (both sample size and number of isotope groups) to verify the accuracy of the parameter recovery even in the presence of intentionally mislabeled isotope groups.

References

1. Paweletz, C. et al. *J Proteome Res* 2010, **9**, 1392–1401.
2. Sietsema, K.; Meng, F.; Yates, N. et al. *Biomarkers* 2010, **15**, 249–258.
3. Neubert, H.; Bonnert, T.; Rumpel, K.; Hunt, B.; Henle, E.; James, I. *J Proteome Res* 2008, **7**.
4. Meng, F.;Wiener, M.; Sachs, J.; Burns, C.; Verma, P.; Paweletz, C.; Mazur, M.; Keyanova, E.; Yates, N.; Hendrickson, R. *J Am Soc Mass Spectrom* 2007, **18**, 226–233.
5. Nittis, T.; Guittat, L.; LeDuc, R.; Dao, B.; Duxin, J.; Rohrs, H.; R.R., T.; Stewart, S. *Mol Cell Proteomics* 2010, **9**, 1144–1156.
6. Keller, A.; Nesvizhskii, A.; Kolker, E.; Aebersold, R. *Anal Chem* 2002, **75**, 4646–4658.
7. Clough, T.; Key, M.; Ott, I.; Ragg, S.; Schadow, G.; Vitek, O. *J Proteome Res* 2009, **8**, 5275–5284.
