# Supplementary material for: Characterization of Serum Proteins Associated with IL28B Genotype among Patients with Chronic Hepatitis C
Source: PLoS One. 2011 Jul 5;6(7):e21854. doi: 10.1371/journal.pone.0021854 (PMC3130042; doi:10.1371/journal.pone.0021854)
Supplement: Table S1 — Protein identification frequency in samples from 41 CHC patients. This table lists each of the proteins identified in the data set along with the proteins' full names and the number of different isotope groups from that protein that were identified. (DOC) [file pone.0021854.s002.doc]

Table S1. Protein identification frequency in samples from 41 CHC patients. This table lists each of the proteins identified in the data set along with the proteins’ full names and the number of different isotope groups from that protein that were identified.

| Uniprot Identifier | Protein Description | Peptides |
| --- | --- | --- |
| APOB_HUMAN | Apolipoprotein B-100 precursor | 500 |
| CO4B_HUMAN | Complement C4-B precursor | 277 |
| CERU_HUMAN | Ceruloplasmin precursor | 165 |
| FINC_HUMAN | Fibronectin precursor | 172 |
| CFAH_HUMAN | Complement factor H precursor | 139 |
| CFAB_HUMAN | Complement factor B precursor | 109 |
| ITIH2_HUMAN | Inter-alpha-trypsin inhibitor heavy chain H2 precursor | 111 |
| PLMN_HUMAN | Plasminogen precursor | 88 |
| CO5_HUMAN | Complement C5 precursor | 91 |
| APOA4_HUMAN | Apolipoprotein A-IV precursor | 108 |
| HEMO_HUMAN | Hemopexin precursor | 113 |
| ITIH4_HUMAN | Inter-alpha-trypsin inhibitor heavy chain H4 precursor | 103 |
| VTDB_HUMAN | Vitamin D-binding protein precursor | 99 |
| AACT_HUMAN | Alpha-1-antichymotrypsin precursor | 72 |
| A1BG_HUMAN | Alpha-1B-glycoprotein precursor | 81 |
| ANT3_HUMAN | Antithrombin-III precursor | 83 |
| KNG1_HUMAN | Kininogen-1 precursor | 68 |
| AFAM_HUMAN | Afamin precursor | 59 |
| ITIH1_HUMAN | Inter-alpha-trypsin inhibitor heavy chain H1 precursor | 64 |
| THRB_HUMAN | Prothrombin precursor | 56 |
| FETUA_HUMAN | Alpha-2-HS-glycoprotein precursor | 76 |
| ANGT_HUMAN | Angiotensinogen precursor | 52 |
| CO7_HUMAN | Complement component C7 precursor | 49 |
| GELS_HUMAN | Gelsolin precursor | 56 |
| APOH_HUMAN | Beta-2-glycoprotein 1 precursor | 54 |
| PGRP2_HUMAN | N-acetylmuramoyl-L-alanine amidase precursor | 42 |
| CO6_HUMAN | Complement component C6 precursor | 45 |
| IC1_HUMAN | Plasma protease C1 inhibitor precursor | 43 |
| VTNC_HUMAN | Vitronectin precursor | 27 |
| CO9_HUMAN | Complement component C9 precursor | 38 |
| ZA2G_HUMAN | Zinc-alpha-2-glycoprotein precursor | 38 |
| FIBA_HUMAN | Fibrinogen alpha chain precursor | 35 |
| CFAI_HUMAN | Complement factor I precursor | 24 |
| FIBB_HUMAN | Fibrinogen beta chain | 41 |
| CO2_HUMAN | Complement C2 precursor | 26 |
| C1R_HUMAN | Complement C1r subcomponent precursor | 17 |
| HEP2_HUMAN | Heparin cofactor 2 precursor | 36 |
| A2AP_HUMAN | Alpha-2-antiplasmin precursor | 25 |
| A2GL_HUMAN | Leucine-rich alpha-2-glycoprotein precursor | 30 |
| AMBP_HUMAN | AMBP protein precursor | 36 |
| CLUS_HUMAN | Clusterin precursor | 37 |
| APOE_HUMAN | Apolipoprotein E precursor | 34 |
| PEDF_HUMAN | Pigment epithelium-derived factor precursor | 38 |
| FIBG_HUMAN | Fibrinogen gamma chain | 32 |
| C1S_HUMAN | Complement C1s subcomponent precursor | 21 |
| LUM_HUMAN | Lumican precursor | 28 |
| THBG_HUMAN | Thyroxine-binding globulin precursor | 27 |
| FA12_HUMAN | Coagulation factor XII precursor | 22 |
| HRG_HUMAN | Histidine-rich glycoprotein precursor | 28 |
| KAIN_HUMAN | Kallistatin precursor | 22 |
| ALS_HUMAN | Insulin-like growth factor-binding protein complex acid labile chain precursor | 23 |
| C4BPA_HUMAN | C4b-binding protein alpha chain precursor | 29 |
| CO8B_HUMAN | Complement component C8 beta chain precursor | 13 |
| SHBG_HUMAN | Sex hormone-binding globulin precursor | 17 |
| CPN2_HUMAN | Carboxypeptidase N subunit 2 precursor | 15 |
| SAMP_HUMAN | Serum amyloid P-component precursor | 20 |
| CO3_HUMAN | Complement C3 precursor | 15 |
| KLKB1_HUMAN | Plasma kallikrein precursor | 11 |
| FCN3_HUMAN | Ficolin-3 precursor | 13 |
| RET4_HUMAN | Retinol-binding protein | 15 |
| ITIH3_HUMAN | Inter-alpha-trypsin inhibitor heavy chain H3 precursor | 9 |
| VWF_HUMAN | von Willebrand factor precursor | 7 |
| C1QC_HUMAN | Complement C1q subcomponent subunit C precursor | 11 |
| CXCL7_HUMAN | Platelet basic protein precursor | 6 |
| CO8A_HUMAN | Complement component C8 alpha chain precursor | 9 |
| CO8G_HUMAN | Complement component C8 gamma chain precursor | 11 |
| A1AT_HUMAN | Alpha-1-antitrypsin precursor | 10 |
| C1QB_HUMAN | Complement C1q subcomponent subunit B precursor | 5 |
| CBPB2_HUMAN | Carboxypeptidase B2 precursor | 5 |
| CHLE_HUMAN | Cholinesterase precursor | 7 |
| TETN_HUMAN | Tetranectin precursor | 11 |
| TSP1_HUMAN | Thrombospondin-1 precursor | 5 |
| PZP_HUMAN | Pregnancy zone protein | 8 |
| PON1_HUMAN | Serum paraoxonase/arylesterase 1 | 9 |
| APOA2_HUMAN | Apolipoprotein A-II precursor | 5 |
| CBG_HUMAN | Corticosteroid-binding globulin precursor | 5 |
| FHR2_HUMAN | Complement factor H-related protein 2 precursor | 8 |
| K1C9_HUMAN | Keratin, type I cytoskeletal 9 | 2 |
| ZC3HD_HUMAN | Zinc finger CCCH domain-containing protein 13 | 3 |
| C1QA_HUMAN | Complement C1q subcomponent subunit A precursor | 5 |
| F13B_HUMAN | Coagulation factor XIII B chain precursor | 2 |
| LBP_HUMAN | Lipopolysaccharide-binding protein | 5 |
| PROS_HUMAN | Vitamin K-dependent protein S precursor | 2 |
| ZPI_HUMAN | Protein Z-dependent protease inhibitor precursor | 4 |
| APOC2_HUMAN | Apolipoprotein C-II | 2 |
| GCC2_HUMAN | GRIP and coiled-coil domain-containing protein 2 | 3 |
| GPX3_HUMAN | Glutathione peroxidase 3 precursor | 3 |
| HBB_HUMAN | Hemoglobin subunit beta | 3 |
| DOPO_HUMAN | Dopamine beta-hydroxylase | 4 |
| B2MG_HUMAN | Beta-2-microglobulin precursor | 2 |
| BTD_HUMAN | Biotinidase precursor | 2 |
| CBPN_HUMAN | Carboxypeptidase N catalytic chain precursor | 2 |
| CO4A_HUMAN | Complement C4-A precursor | 4 |
| FA10_HUMAN | Coagulation factor X precursor | 2 |
| FETUB_HUMAN | Fetuin-B precursor | 2 |
| LG3BP_HUMAN | Galectin-3-binding protein precursor | 2 |
| QSOX1_HUMAN | Sulfhydryl oxidase 1 precursor | 3 |
| SEPP1_HUMAN | Selenoprotein P precursor | 2 |
| PRAM_HUMAN | PML-RARA-regulated adapter molecule 1 | 3 |
| APOC3_HUMAN | Apolipoprotein C-III precursor | 2 |
| CD14_HUMAN | Monocyte differentiation antigen CD14 precursor | 2 |
| NUDT5_HUMAN | ADP-sugar pyrophosphatase | 2 |
| PRG4_HUMAN | Proteoglycan-4 precursor | 2 |
| BGH3_HUMAN | Transforming growth factor-beta-induced protein ig-h3 | 2 |
| CC155_HUMAN | Coiled-coil domain-containing protein 155 | 2 |
| CENPF_HUMAN | Centromere protein F | 2 |
| F13A_HUMAN | Coagulation factor XIII A chain | 2 |
| NSD2_HUMAN | Probable histone-lysine N-methyltransferase NSD2 | 2 |
| PXDNL_HUMAN | Peroxidasin-like protein | 2 |
| RPGR_HUMAN | X-linked retinitis pigmentosa GTPase regulator | 2 |
